# Comparative Analysis of Phylogenetic Assignment of Human and Avian ExPEC and Fecal Commensal *Escherichia coli* Using the (Previous and Revised) Clermont Phylogenetic Typing Methods and its Impact on Avian Pathogenic *Escherichia coli* (APEC) Classification

**DOI:** 10.3389/fmicb.2017.00283

**Published:** 2017-02-23

**Authors:** Catherine M. Logue, Yvonne Wannemuehler, Bryon A. Nicholson, Curt Doetkott, Nicolle L. Barbieri, Lisa K. Nolan

**Affiliations:** ^1^Department of Veterinary Microbiology and Preventive Medicine, Iowa State University, AmesIA, USA; ^2^Department of Statistics, North Dakota State University, FargoND, USA

**Keywords:** phylogenetic typing, *Escherichia coli*, extraintestinal pathogenic *E. coli* (ExPEC), avian pathogenic *E. coli* (APEC), classification

## Abstract

The Clermont scheme has been used for subtyping of *Escherichia coli* since it was initially described in early 2000. Since then, researchers have used the scheme to type and sub-type commensal *E. coli* and pathogenic *E. coli*, such as extraintestinal pathogenic *E. coli* (ExPEC), and compare their phylogenetic assignment by pathogenicity, serogroup, distribution among ExPEC of different host species and complement of virulence and resistance traits. Here, we compare assignments of human and avian ExPEC and commensal *E. coli* using the old and revised Clermont schemes to determine if the new scheme provides a refined snapshot of isolate classification. 1,996 *E. coli* from human hosts and poultry, including 84 human neonatal meningitis *E. coli* isolates, 88 human vaginal *E. coli*, 696 human uropathogenic *E. coli*, 197 healthy human fecal *E. coli*, 452 avian pathogenic *E. coli* (APEC), 200 retail poultry *E. coli*, 80 crop and gizzard *E. coli* from healthy poultry at slaughter and 199 fecal *E. coli* from healthy birds at slaughter. All isolates were subject to phylogenetic analysis using the Clermont et al. (2000, 2013) schemes and compared to determine the effect of the new classification on strain designation. Most of the isolates’ strain designation remained where they were originally assigned. Greatest designation change occurred in APEC where 53.8% of isolates were reclassified; while classification rates among human strains ranged from 8 to 14%. However, some significant changes were observed for UPEC associated strains with significant (*P* < 0.05) designation changes observed from A to C and D to E or F phylogenetic types; a similar designation change was noted among NMEC for D to F designation change. Among the APEC significant designation changes were observed from A to C and D to E and F. These studies suggest that the new scheme provides a tighter and more meaningful definition of some ExPEC; while the new typing scheme has a significant impact on APEC classification. A comparison of phylogenetic group assignment by content of virulence, resistance, replicon and pathogenicity island genes in APEC suggests that insertion of pathogenicity islands into the genome appears to correlate closely with revised phylogenetic assignment.

## Introduction

*Escherichia coli* are common inhabitants of the intestinal tracts of mammals and one of the most extensively studied bacteria worldwide. They may inhabit a host as harmless symbionts, or depending on their panoply of virulence traits and/or certain predisposing conditions, they may be responsible for both intestinal and extra-intestinal disease. Pathogenic *E. coli* may be subdivided into Intestinal Pathogenic *E. coli* (InPEC) or Extraintestinal Pathogenic *E. coli* (ExPEC) pathotypes depending on their host target tissue and disease syndrome they cause. Subpathotypes of these pathotypes exist depending on the organ system they target, the pathogenic mechanisms they employ and/or the host species in which they cause disease. Each grouping tends to possess certain common traits, which allow them to survive, grow and cause disease in their target location. For example, the ExPEC causing meningitis in human newborns tends be of certain serogroups, in particular O18 (Logue et al., 2012; Nicholson et al., 2016a,b). They also tend to possess large virulence plasmids and K1 capsular antigen (Rodriguez-Siek et al., 2005a). Human uropathogenic *E. coli* (UPEC) tend to be of the O2 and O6 serogroups and lack virulence plasmids (Rodriguez-Siek et al., 2005a). Similarly, Avian Pathogenic *E. coli* (APEC), the ExPEC causing colibacillosis in birds, are typified by possession of large virulence plasmids (Rodriguez-Siek et al., 2005b; Barnes et al., 2008; Johnson et al., 2008a), and although there is great diversity in the serogroups of APEC, the more common ones reported are O1, O2, and O78 (Rodriguez-Siek et al., 2005b; Nolan et al., 2013).

Herzer et al. (1990) and Selander et al. (1987) used multilocus enzyme electrophoresis (MLEE) to produce a topology for the ECOR collection which classified *E. coli* in the ECOR collection into four main groups, identified as A, B1, B2, and D and an outlier group that could not be grouped (UG). Work by Lecointre et al. (1998) attempted phylogenetic analysis based on the work of Herzer and noted that A and B1 groups appeared to be sister groups while B2 were considered to contain the highly virulent extra-intestinal strains. In early 2000, Clermont et al. (2000) revised the typing scheme using a simple triplex polymerase chain reaction (PCR) for assigning *E. coli* into phylogenetic groups. This scheme focused on two genes and an anonymous DNA fragment: (1) *chuA*, a gene associated with heme transport in enterohemorrhagic *E. coli* (EHEC); (2) *yjaA*, a gene of unknown function; and (3) an anonymous DNA fragment called TSPE4.C2 that was later identified as a putative lipase esterase gene (Gordon et al., 2008). The method was found to show high correlation with other reference methods including multi-locus sequence type (MLST) analysis (Gordon et al., 2008). Over the years, hundreds, if not thousands of researchers, have taken advantage of this tool for use in their analysis of *E. coli* including the authors of this paper (Rodriguez-Siek et al., 2005a,b; Johnson et al., 2008a,b, 2009; Logue et al., 2012; Hussein et al., 2013). In early work, where the *E. coli* phylogenetic group was described (Clermont et al., 2000), authors reported that ExPEC associated with human disease belonged primarily to the group B2 phylogenetic group and to a lesser extent to group D (Picard et al., 1999; Johnson and Stell, 2000), while most commensal strains appeared to belong to group A. However, Avian Pathogenic *E. coli*, the ExPEC of poultry, appeared to differ significantly in their phylogenetic assignment with the majority of APEC being assigned to groups A and D and less than 20% assigned to group B2 (Rodriguez-Siek et al., 2005a).

Despite such distinguishing traits among the majority of *E. coli* representing the ExPEC subpathotypes, considerable overlap among them do exist (Johnson et al., 2008b, 2012a). Indeed, subsets of the *E. coli* from each of these ExPEC subpathotypes have been found to overlap in serogroup, virulence genotype, and ability to cause disease in animal models, lending credence to the hypothesis that some ExPEC are zoonotic (Johnson et al., 2008b, 2012a; Tivendale et al., 2010). Such overlapping ExPEC also tend to fall into the B2 phylogenetic group, an assignment common to human ExPEC, but less frequent in pathogenic *E. coli* of poultry (Rodriguez-Siek et al., 2005a).

Here, we reconsider the relationship of ExPEC’s phylogenetic assignment to zoonotic potential and frank pathogenicity following a comparative analysis of an extensive collection of *E. coli* from healthy and diseased human and avian hosts using a revised phylogenetic typing method (Clermont et al., 2013). It is our hypothesis that this more exacting scheme will better distinguish APEC from human ExPEC and more clearly delineate ExPEC from commensals. The recently described Clermont revision (Clermont et al., 2013) also provides an opportunity to determine if we can enhance the resolution and discrimination between avian and human strains and gain insight into their pathogenicity using the newer scheme which has been expanded from assignment to groups A, B1, B2, and D to include phylogenetic types A, B1, B2, C, D, E, and F, which can accommodate the *E. coli sensu stricto* types and additional clades that include some of the more cryptic strains (Walk et al., 2009; Clermont et al., 2011a).

The association between pathogenic traits of ExPEC and their virulence gene repertoire has been previously explored in some of our earlier work (Johnson et al., 2012a,b; Logue et al., 2012); however, the potential of pathogenic traits associated with phylogenetic types warrants consideration into the potential role of virulence genes and their linkage with specific phylogenetic types. The purpose of this study was to compare assignment of the phylogenetic type in an extensive collection of ExPEC including both human and animal ExPEC strains and assess the usefulness of the phylogenetic typing tools in subtyping various ExPEC collections. In addition, the role of phylogenetic type in APEC was examined to assess the association between virulence gene carriage and phylogenetic type.

## Materials and Methods

### Strains Used in the Study

A total of 1996 strains of *E. coli* were included in this study consisting of 84 strains associated with neonatal meningitis (designated as NMEC), these have been described elsewhere but consist of strains isolated from neonatal meningitis cases in the US and Netherlands (Johnson et al., 2002; Logue et al., 2012). An additional 88 strains of human vaginal *E. coli* (HVEC) (Obata-Yasuoka et al., 2002; Johnson et al., 2012a). Also included were 696 isolates associated with human urinary tract infections (UPEC), including 176 from urinary tract infections (UTIs) in children; 40 from urosepsis; 168 associated with pyelonephritis; 81 associated with cystitis; and the remaining 231 from what were identified as general UTIs. Included among the human strains tested are 197 human fecal *E. coli* (HFEC) recovered from healthy volunteers (Logue et al., 2012).

Among the avian *E. coli* collection are 452 isolates of *E. coli* recovered from the lesions of poultry clinically diagnosed with colibacillosis. These Avian Pathogenic *E. coli* (APEC) were isolated from chickens, turkeys, geese, ducks etc. afflicted with colisepticemia, airsacculitis, perihepatitis, swollen head syndrome, omphalitis, and other such colibacillosis syndromes; these strains have been described previously in studies from our lab (Rodriguez-Siek et al., 2005b; Johnson et al., 2008b). An additional 80 isolates from the crops (40) and gizzards (40) of healthy turkeys (CGEC) (Johnson et al., 2009); 200 isolates recovered from retail poultry meat (RPEC) (Johnson et al., 2009), and 199 *E. coli* from the feces of apparently healthy poultry (AFEC) are included in this study (Johnson et al., 2009).

### Preparation of DNA Template

All isolates were removed from frozen stocks and recovered to MacConkey (MAC) agar with incubation overnight at 37°C. Single isolated colonies were picked and incubated in 1 ml of Luria Bertani broth (LB) at 37°C overnight, and the culture used for preparation of boil preps. Overnight cultures were centrifuged at 13,000 × *g* for 2 min to precipitate the bacteria, and the supernatant removed and discarded. The remaining pellet was re-suspended in 200 μl of sterile deionized water and heated on a heating block for 10 min at 100°C. Following heating, the suspension was centrifuged again to remove cellular debris, and the supernatant removed to a new tube as DNA stock. All DNA was stored frozen at -20°C until use.

### Phylogenetic Typing PCR Protocols

Samples of the DNA stock was subjected to PCR amplification following the phylogenetic protocol first described by Clermont et al. (2000). PCR amplification used a 25 μl volume containing 10X PCR buffer, 20 p mol of each primer, and 2.5 U of Taq polymerase with 1-2 μl of genomic DNA from the boil prep. PCR amplifications were carried out in a thermocycler (Eppendorf, Germany) using the following amplification conditions: denaturation for 5 min at 94°C followed by 30 cycles of 30 s at 94°C; 30 s at 55°C and 30 s at 72°C with a final extension at 72°C for 7 min. Primer pairs used are described in **Table 1**.

**Table 1:** Primers used in the phylogenetic typing PCR assays described by Clermont et al. (2000, 2013).

	Primer name		Product size	Reference
Quadruplex (Old)	*chuA.1*	GACGAACCAACGGTCAGGAT	279 bp	Clermont et al., 2000
	*chuA.2*	TGCCGCCAGTACCAAAGACA		
	*yjaA.1*	TGAAGTGTCAGGAGACGCTG	211 bp	Clermont et al., 2000
	*yjaA.2*	ATGGAGAATGCGTTCCTCAAC		
	*TspE4C2.1*	GAGTAATGTTCGGGGCATTCA	152 bp	Clermont et al., 2000
	*TspE4C2.2*	CGCGCCAACAAAGTATTACG		
Quadruplex (New)	*chuA.1b*	ATGGTACCGGACGAACCAAC	288	Clermont et al., 2013
	*chuA.2*	TGCCGCCAGTACCAAAGACA		Clermont et al., 2000
	*yjaA.1b*	CAAACGTGAAGTGTCAGGAG	211	Clermont et al., 2013
	*yjaA.2b*	AATGCGTTCCTCAACCTGTG		
	*TspE4C2.1b*	CACTATTCGTAAGGTCATCC	152	Clermont et al., 2013
	*TspE4C2.2b*	AGTTTATCGCTGCGGGTCGC		
	*AceK F*	AACGCTATTCGCCAGCTTGC	400	Clermont et al., 2013
	*ArpA1 R*	TCTCCCCATACCGTACGCTA		Clermont et al., 2004
Group E	*ArpAgpE F*	GATTCCATCTTGTCAAAATATGCC	301	Lescat et al., 2013
	*ArpAgpE R*	GAAAAGAAAAAGAATTCCCAAGAG		
Group C	*trpAgpC.1*	AGTTTTATGCCCAGTGCGAG	219	Lescat et al., 2013
	*trpagpC.2*	TCTGCGCCGGTCACGCCCC		
Internal Cont	*trpBA F*	CGGCGATAAAGACATCTTCAC	489	Clermont et al., 2008
	*trpBA R*	GCAACGCGGCCTGGCGGAAG		

Samples of the DNA stock were also subjected to phylogenetic typing using the revised protocols described by Clermont et al. (2013). Here a 25 μl PCR reaction volume as described above with the following PCR conditions: denaturation for 4 min at 94°C followed by 30 cycles of 5 s at 94°C; 30 s at 64°C (group E), or 63°C (quadruplex) or 66°C (group C) and 30 s at 72°C with a final extension at 72°C for 5 min.

PCR products generated for the two schemes were subjected to electrophoresis in 2% (w/v) agarose gels in 1X TAE buffer and run at 120 V for 2 h. A Hi-Lo molecular weight marker (100 bp; Minnesota Molecular, Minneapolis, MN, USA) was used as the size standard; negative (DNAse/RNAse free water) and positive control strains (ATCC and the ECOR reference collection) were included in each gel as appropriate. Gels were stained in ethidium bromide, and bands corresponding to each gene present were recorded using a UV Imager (Omega Fluor, Aplegen, Pleasanton, CA, USA).

### Virulence Genotyping, Plasmid Replicon and Resistance Typing of APEC and AFEC

Test and control organisms were examined using multiplex PCR for 208 traits, including chromosomally located ExPEC virulence genes, including some of their allelic variants; genes associated with the pathogenicity islands (PAIs) of ExPEC virulence plasmids, genes of unknown function found in genomic islands of ExPEC isolate APEC O1 (Johnson et al., 2007a, 2012b); and various plasmid replicons (see Supplementary Table 1 for primer sequences). The approach used in this analysis has been described by Logue et al. (2012) using multiplex panels that have been previously described and detailed (Rodriguez-Siek et al., 2005a; Johnson et al., 2006a,b,c, 2007b). All primers were obtained from Integrated DNA Technologies (Coralville, IA, USA). PCR was performed as previously described (Rodriguez-Siek et al., 2005a). Strains known to possess or lack the genes of interest were examined with each amplification procedure as controls. Reactions were performed twice. An isolate was considered to contain the target of interest if it produced an amplicon of the expected size.

### Biostatistics

All data generated from the identification of phylogenetic types by the two typing schemes was examined using non-parametric tests and input into spreadsheets and compared using the Wilcoxoan–Mann–Whitney Test (SPSS, IBM Corp, Armonk, NY, USA). Statistical significance was accepted at *P* < 0.05.

A chi-square test of homogeneity was used for comparison between groups, and Fisher’s exact test was used where the assumptions of the chi-square test did not hold (Snedecor and Cochran, 1989). In a further attempt to discern patterns among all isolates based on their content of virulence genes, a linear discriminant analysis (LDA) was used to determine if an isolate type (APEC or AFEC) could be predicted based on the virulence genes present (Huberty, 1994). Although the use of data from binary variables in an LDA, as done here, violates the assumption of multivariate normality, LDA was used since parametric LDA can be very robust in spite of such violations (McLachlan, 1992).

**FIGURE 1:**
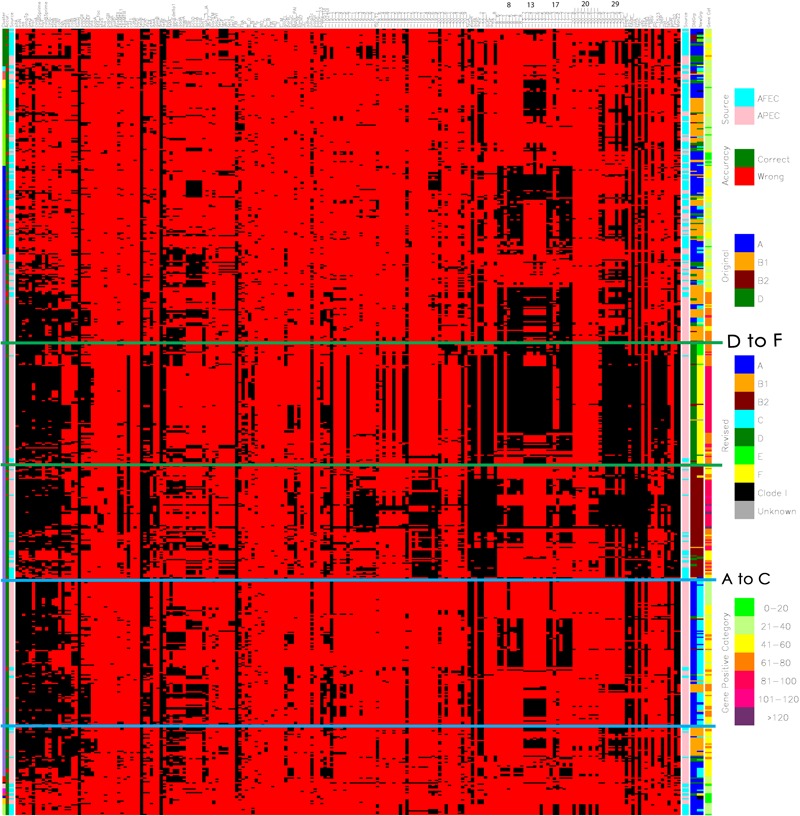
**Diagram merging the cluster analysis and linear discriminant analysis (LDA) of the genotyping results of avian pathogenic *E. coli* (APEC) and avian fecal *E. coli* (AFEC) strains.** The first row (the uppermost row at the top of the figure) identifies the genes that were screened for using multiplex PCR reaction a total of 208 genes are included here as described previously (Logue et al., 2012). The first column on the left indicates the numbers of clusters that the strains were located to – there are 15 indicated (1) Green; (2) Light blue; (3) Pink; (4) Yellow; (5) Lime green; (6) Dark blue; (7) Purple; (8) Red; (9) Black; (10) Salmon pink; (11) Magenta; (12) Orange; (13) Navy blue; (14) Dark yellow; (15) Light lime green. The second column shows the accuracy of prediction from the linear discriminant analysis (LDA) of an isolate type based on possession of genes/traits. Green indicates a correct prediction as to whether an isolate is an APEC or an AFEC strain, whereas red indicates a misprediction. The third column indicates the source of the isolates, where AFEC strains are denoted as light blue, and APEC strains are identified in salmon pink. The next set of columns of red and black bars shows the results of PCR for a single gene or trait. The identity of each gene tested is shown above the column at the far right of the diagram. In the body of the figure, a black line means that the gene of interest is present in a particular isolate, whereas a red line means the gene is absent. The column following the black and red pattern shows the source of the isolates (same as the third column). Column 206 (made up of brown, green, blue, and orange colors) indicates the phylogenetic groups of each isolate using the Clermont 2000 scheme: blue, phylogenetic group A; orange, B1; brown, B2; and green, D. Column 207 (made up of blue, orange, brown, light blue, green, lime green, yellow, black, and gray) indicates the phylogenetic groups of each isolate using the Clermont 2013 scheme: blue, phylogenetic group A; orange, B1; brown, B2; and light blue. C; green, D; lime green E; yellow, F; black, clade 1; gray, unknown. Column 208 indicates the indicates the gene/trait content for each strain, where isolates in category 1 contain 1 to 20 of the tested genes/traits and are identified as lime green; category 2 isolates are identified in light lime green and possess 21 to 40 genes; category 3 isolates are identified in yellow and possess 41 to 60 genes; category 4 isolates are identified in orange and possess 61 to 80 genes; and category 5 isolates are identified in red and possess 81 to 100 genes. Category 6 isolates are identified in magenta and possess 101–120 genes while category 7 identifies isolates in purple possessing > 120 genes. Above the columns on the right side of the image some of the pathogenicity islands have been indicated by the numbers 8, 13, 17, 20, and 29. Detail view available at: https://figshare.com/s/550bc7f2b3eadd69889e.

Additionally, a cluster analysis of the APEC and AFEC isolates was performed using the average linkage method based upon Jaccard’s dissimilarity coefficient calculated from the presence of all genes analyzed (SAS 9.22) (Anonymous, 2010). In order to better discern patterns among the isolates, results of the cluster and discriminant analyses and the isolates’ virulence genotypes and phylogenetic groups were used to construct a single figure based on the principles of Eisen et al. (1998) (see **Figure 1**).

## Results

All strains were analyzed individually using both Clermont protocols. The data generated was subjected to comparative analysis to determine the effect of the change in type using the newer protocol. **Table 2** shows the assignment and distribution of pathogenic and commensal *E. coli* strains by the old and revised phylogenetic typing schemes. Overall, the majority of human isolates remained in the same assigned types, while over half of the avian isolates were reassigned to new categories. B2 and D assignments remained the dominant assignments among the human ExPEC. Most UPEC strains were typed as B2 and D by the older scheme and these two types still dominated in the revised scheme but not all classified equally by the two typing methods with a considerable number of A and D strains reclassifying to the newer types B2, C, E, and F with significant redistribution (*P* < 0.05) noted for A to C and D to E and F classification (see Supplementary Table 2).

**Table 2:** Classification assignment (phylogenetic type) of *E. coli* using the original and revised Clermont schemes.

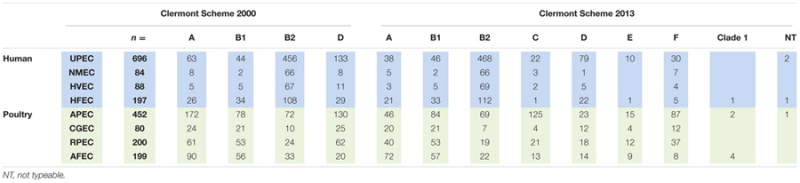

For the NMEC strains, the majority resided in the B2 type using the old typing scheme and similar results were observed using the revised scheme. However, there was a significant (*P* < 0.05) redistribution for the A to C and D to F grouping (see Supplementary Table 3).

For the other human strains (HVEC), B2 and D were the most common categories of isolates by the older scheme but this pattern became diluted using the newer typing scheme with B1, B2, and D dominating but significant changes (*P* < 0.05) were only observed in the A to C and D to F categories (see Supplementary Table 4); For the human fecal strains (HFEC) the dominant categories by the old typing scheme were B1 and B2 and in the revision these two categories still dominated; significant changes were noted for A to C and D to F (*P* < 0.05) (see Supplementary Table 5).

When the avian isolates were examined, the revised phylogenetic typing scheme appeared to result in a more refined classification of the isolates. Here, APEC underwent significant reassignment (*P* < 0.05) among isolates classified in the A and D categories with reclassification of A to C and D to E and F phylogenetic groups. Similar strain classification changes were observed for isolates from the crops and gizzards of healthy birds [CGEC from A to C and D to F (*P* < 0.05)]; while reclassification among retail poultry meat was significant in the A to C (*P* < 0.05) classification and isolates from the feces of apparently healthy birds identified significant (*P* < 0.05) reclassification in the A to C and D to E and F groups (see Supplementary Tables 6–9).

A drill down comparison of the changes that occurred among the phylogenetic groups found that overall approximately 25.6% of all isolates examined became re-classified using the revised Clermont scheme (**Table 3**) with about 13.05% overall re classification among the human strains and 40.49% among the poultry associated strains. Overall rates of change were found to range from 0.1% to levels of 9.5%. The most significant changes occurred for two type changes: A to C where 9.57% of the collection showed this re-classification and D to F where 9.21% of the entire collection was re-classified. When the overall rate of re-classification was examined at the source level among the human isolates, 8–13% of the UPEC, NMEC, HVEC, or HFEC were reclassified compared to the animal sources where reclassification rates ranged from 21.6 to 53.3% with the greatest rate of reclassification observed for more than half of all APEC examined. Among the human strains greatest reclassification was noted for A to C (2.72%); D to B2 (2.44%) and D to F (4.23%). In contrast, change rates among poultry were greatest in A to C (71.4%) and D to F (14.93%).

**Table 3:** Changes in Phylogenetic type assignment based on application of the new phylogenetic typing scheme to original assignment.

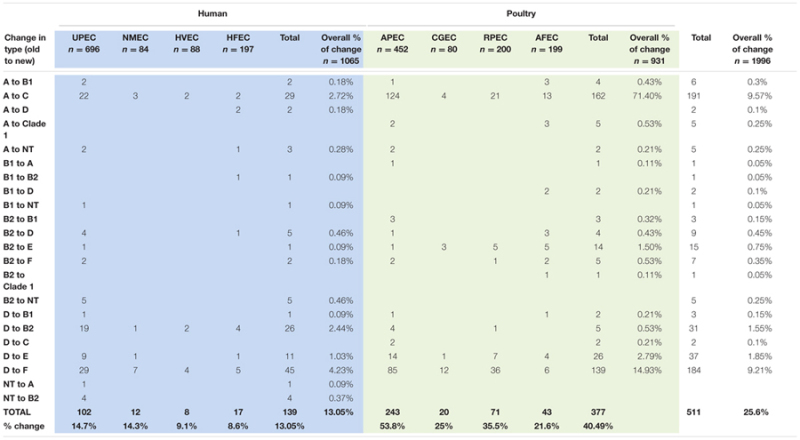

A further analysis of the cause of the high rate of reclassification in APEC required a full virulence, resistance, replicon, and pathogenicity island gene analysis of the strains used in the study using a cluster analysis. The prevalence of all genes was assessed by comparing APEC with the AFEC strains. **Figure 1** shows the cluster analysis of virulence, resistance, replicon and island genes for all APEC and AFEC used in the study. A detailed observation of where phylogenetic type changes from A to C and D to F are evident in the lower sections of the figure and are indicated by boundary boxes in blue and green (**Figure 1**). When examined in detail to determine potential influences on the reclassification based on genes present, it became evident that APEC isolates that converted from D to F had a greater prevalence of selected genomic islands; these include islands 29, 17, 13, and 8. Of interest, those strains that identified as D and remained as D after re-analysis were more likely to have a lower prevalence of island associated genes and the overall gene count was significantly lower (40–60 genes compared with the re-assigned isolates that harbor > 100 genes). Also of interest those isolates identified as D by the two phylogenetic typing methods tended to be located randomly in the analysis and not form a distinct cluster with random distribution observed among the other phylogenetic types, in particular the A and B types.

The second cluster with revised designation of A to C were more likely to possess *fim, irp2, eae*, and *fepC* genes as well as those possessing genomic islands 8 and 17 while a second core group possessed genomic island 13. Most of the strains in this cluster possessed 21–80 genes. Those isolates that did not transition to C but were identified as A and remained so tended to cluster in the bottom quadrant of the diagram along with AFEC strains and possessed low numbers of genes (range 21–60). While a second group of A strains can be found in the upper region of the diagram among the A and B1 strains and also typically possessed low numbers of genes (<40).

## Discussion

The focus of this study was to assess the impact of the new Clermont scheme (2013) on assignment of phylogenetic types to *E. coli* of different pathotypes and origin. Changes in the designation of phylogenetic type are important in understanding the accuracy of the phylogenetic typing method and in identifying new groups of emerging strains that are better designated or identified as a result of this analysis.

In this study, most of phylogenetic types identified by the new scheme resulted in significant re-distribution of identified isolates into new groups from their original designation. This was particularly evident among the human isolates where a considerable number of UPEC isolates were reclassified from A to C and D to E or F (**Tables 2** and **3**; Supplementary Table 2). These changes were significant at the *P* < 0.05 level. For the other human stains (NMEC, HVEC, and HFEC) significant (*P* < 0.05) differences were also noted for A to C and D to F changes in designation (Supplementary Tables 3–5).

When examined for avian strains, the changes in designation were similar to those seen with the human strains but a considerable number of APEC changed designation (>50% of the collection). The most significant changes were observed for conversion from A to C (*P* < 0.05) and D to E or F (*P* < 0.05) (Supplementary Table 6). These similar re-classification patterns were also observed for CGEC, RPEC, and AFEC but at much overall lower prevalence levels (**Tables 2** and **3**; Supplementary Tables 7–9).

For most part, the current study appeared to verify that the expanded phylogenetic typing scheme was accurate and about 75% of isolates identified in the original typing scheme retained their original phylogenetic type. However, about 25% of the overall collection was assigned to a different phylogenetic types not previously recognized and among those, about 22.7% of the isolates moved into new designations not recognized in the earlier scheme (i.e., C, E, F, and clade 1) (**Table 3**) with biggest changes noted in the APEC collection.

Rates of change in the human isolates ranged from 8 to 13% with the highest rates of change observed in UPEC and NMEC. Of these the highest rates of change were observed in A to C designation (old to new) and D to F. From the avian isolates the rates of change in designation were greater with change rates ranging from 21 to 53%. Greatest rates of change were observed for the APEC and RPEC designated strains. Most significantly, the greatest rates of change were observed in the A to C and D to F designations. Understanding what these definitions and change in type mean is a challenge and there is limited information as to what factors drive the grouping schemes.

Lecointre et al. (1998) identified A and B1 phylogenetic types as sister groups, being different in their ecological niches with the ability to exploit different characteristics. In this study, most of our A and B1 isolates appeared to cluster together with equal distribution among APEC and AFEC. The level of gene carriage was also relatively low with most having 20–40 genes although some had greater than 60 genes present. Aside from the group of isolates that re-classified, the A phylogenetic types to C this observation would appear to be relatively accurate for the APEC and AFEC collection analyzed and isolates characterized as A or B1 appeared to have low pathogenic potential as well as a low prevalence of pathogenicity, resistance and island associated genes.

In a paper prior to the Clermont revision, Carlos et al. (2010) identified some new variants of the phylogenetic type using the terms A_0_, B2_3_, D_1_ and D_2_ as sub-types of the original phylogenetic types. The method used in their approach would, however, appear to be limited when compared with our study where some of these subtypes were suggested to be only found in human strains – and its presence elsewhere would suggest human fecal contamination – this was not the case in our study and a considerable number of B2_3_ could be found among our APEC collection when their profiling approach was used and it is unlikely that there was human fecal strains associated with production birds supporting our suggestion that the scheme has limitations in its application. Another concern with the (Carlos et al., 2010) analysis may be related to the small collection of stains used and their source which may also limit the generation of a meaningful analysis (a total of 241 isolates were examined compared to 1,996 in the current study and more than 1,000 in the Clermont 2013 study) and none of the strains appear to be from a disease state in the identified hosts which may also limit analysis for certain phylogenetic types which are more associated with pathogenesis and are likely more virulent than fecal isolates alone.

Using the new phylogenetic analysis approach, this study was able to re-assign a significant number of APEC that appeared to misidentify as D using the earlier typing scheme but were reclassified as F due to the incorporation of the *arpA* gene in the refined analysis. Of interest, this group of strains also harbored a significant number of virulence, resistance, replicon and pathogenicity island associated genes. The Clermont 2013 paper (Clermont et al., 2013) does not indicate what influences the re-classification aside from the inclusion of the *arpA* gene. In the most recent Clermont paper (Clermont et al., 2013) the authors suggest that strains assigned to the phylogenetic types A and D from the original scheme may need to be re-assessed using the newer scheme. Based on the analysis of strain typing in this study, the authors of this paper recommend revisiting the typing of A and D isolates in an effort to tease out some of the more pathogenic strains that may otherwise be overlooked. In the current study, re-classification had a significant impact on APEC strains and some of the human UPEC. The challenge in light of the re-classification was to determine potential factors that may have an influence on the change in designation. Most of the papers by Clermont et al. (2013) have examined the influence of genes on the classification of isolates into the phylogenetic groups using sequence and MLST contributing to the assignments. The current study took the analysis a little further and examined the collection of APEC and AFEC for 208 virulence and resistance associated genes to assess the potential role of virulence traits on strain designation. Of interest, included in the analysis were gene data from genomic islands of APEC O1 that have also been described in other ExPEC (Johnson et al., 2012b). The green bordered box in **Figure 1** highlights the group of APEC that changed designation from D to F, of note in this collection are a considerable number of pathogenicity island genes designated as genomic island (GI) 29 (currently of unknown function), GI 17 (prophage), GI 13 (prophage and ExPEC Island containing *cdt* locus) and GI 8 (prophage), GI 22 which includes the genes identified as *eae* (intimin attachment), and *auf* (associated with fimbriae) (Buckles et al., 2004; Johnson et al., 2012b). Relatively little is known about the role of these genomic islands in APEC but previous studies would suggest that these genes are virulence associated and occur at a higher prevalence in APEC than other ExPEC although some of these islands have been found in other ExPEC not of avian origin (Johnson et al., 2012b; Logue et al., 2012). Of significance in this study is that strains that became reclassified as F from their original D designation using the new Clermont scheme appeared to harbor higher levels of pathogenicity, resistance, replicon and pathogenicity island-associated genes and that the new designation would appear to indicate that APEC designated as the F phylogenetic type are probably highly pathogenic. Also of note these “converted” strains lie next to the B2 phylogenetic group, and belong in the same cluster further supporting their pathogenic potential; an observation that Clermont et al. (2013) suggested that phylogenetic type F are a sister group to the phylogenetic group B2 (Jaureguy et al., 2008; Clermont et al., 2011b) and may have been a branch of B2 (Gordon et al., 2008), while in their study Gordon et al. (2008) noted that the Clermont method identified a number of strains as D (old scheme) that appeared to have a B2 phylogenetic type if analysis was based on MLST data. To confirm that the F phylogenetic type is truly a collection of highly pathogenic strains of APEC, further work using animal models is warranted. Regardless, this is the first study to identify this phylogenetic type as a pathogenic type in avian associated ExPEC and the link that pathogenicity islands may play in influencing the pathotype.

A second cluster of isolates that changed designation among APEC was the A to C group. This group of isolates is identified in **Figure 1** as surrounded by the blue box lines. Most isolates in this designation change were of lower virulence, resistance, replicon and pathogenicity island gene content ranging from 21 to 60 genes with GI 17 (prophage), and GI 8 (prophage) genes being present in about half of the isolates in this group while one third of the group appeared to possess GI 13 – a 12 kb island containing putative adhesion/attaching and effacing genes with similarity to the *eae* gene of diarrheagenic *E. coli* (Tzipori et al., 1995; Johnson et al., 2012b). The definition of this group is not as definitive as the previously described phylogenetic F group as within the group there are at least two different profiles: those possessing GI 17 and 8 and those possessing GI 13. In addition, the gene count would appear to be slightly lower (range 21–60 genes) suggesting these may be of lower pathogenic potential, and validation would warrant animal studies. However, this group is also located in the same cluster as the D to F and B2 designated strains. Clermont et al. (2011b) suggests that isolates of phylogenetic type C may be closely related to B1 but are distinct. This observation also fits with the current study as some of our C designated strains occupy the same cluster as B1 strains and also appear to have an overall lower gene count.

## Conclusion

Use of the Clermont typing schemes has greatly enhanced our classification of ExPEC from human and animal hosts. The identification of genomic islands as important factors in the pathogenicity of APEC and other ExPEC is valuable in determining the pathogenic potential of strains under investigation. This study serves to advance our knowledge of the Clermont phylogenetic typing scheme and enhances our studies of APEC by identification of virulence factors and traits that may be responsible for the clustering of certain phylogenetic types.

## Author Contributions

Conceived and designed the experiments: CL and LN. Performed the experiments: YW, BN, and NB. Analyzed the data: CL, CD, LN, and NB. Contributed reagents/materials/analysis tools: CL, CD, and LN. Wrote the paper: CL and LN.

## Conflict of Interest Statement

The authors declare that the research was conducted in the absence of any commercial or financial relationships that could be construed as a potential conflict of interest.

The reviewer VG and handling Editor declared their shared affiliation and the handling Editor states that the process nevertheless met the standards of a fair and objective review.

## References

[B1] Anonymous (2010). *SAS/STAT(R) 9.22 User’s Guide.* Cary, NC: SAS Institute Inc.

[B2] Barnes H. J. Nolan L. K. Vaillancourt J. P. (2008). “Colibacillosis,” in *Diseases of Poultry* 12th Edn eds Saif Y. M. Fadly A. M. Glisson J. R. McDougald L. R. Nolan L. K. Swayne D. E. (Ames, IA: Blackwell Publishing) 691–732.

[B3] Buckles E. L. Bahrani-Mougeot F. K. Molina A. Lockatell C. V. Johnson D. E. Drachenberg C. B. (2004). Identification and characterization of a novel uropathogenic *Escherichia coli*-associated fimbrial gene cluster. *Infect. Immun* 72 3890–3901. 10.1128/IAI.72.7.3890-3901.200472/7/3890 15213132 PMC427398

[B4] Carlos C. Pires M. M. Stoppe N. C. Hachich E. M. Sato M. I. Gomes T. A. (2010). *Escherichia coli* phylogenetic group determination and its application in the identification of the major animal source of fecal contamination. *BMC Microbiol.* 10:161 10.1186/1471-2180-10-161 PMC2889953 20515490

[B5] Clermont O. Bonacorsi S. Bingen E. (2000). Rapid and simple determination of the *Escherichia coli* phylogenetic group. *Appl. Environ. Microbiol.* 66 4555–4558. 10.1128/AEM.66.10.4555-4558.2000 11010916 PMC92342

[B6] Clermont O. Bonacorsi S. Bingen E. (2004). Characterization of an anonymous molecular marker strongly linked to *Escherichia coli* strains causing neonatal meningitis. *J. Clin. Microbiol.* 42 1770–1772. 10.1128/JCM.42.4.1770-1772.2004 15071045 PMC387582

[B7] Clermont O. Christenson J. K. Denamur E. Gordon D. M. (2013). The Clermont *Escherichia coli* phylo-typing method revisited: improvement of specificity and detection of new phylo-groups. *Environ. Microbiol. Rep.* 5 58–65. 10.1111/1758-2229.12019 23757131

[B8] Clermont O. Gordon D. M. Brisse S. Walk S. T. Denamur E. (2011a). Characterization of the cryptic *Escherichia* lineages: rapid identification and prevalence. *Environ. Microbiol.* 13 2468–2477. 10.1111/j.1462-2920.2011.02519.x 21651689

[B9] Clermont O. Lescat M. O’Brien C. L. Gordon D. M. Tenaillon O. Denamur E. (2008). Evidence for a human-specific *Escherichia coli* clone. *Environ. Microbiol.* 10 1000–1006. 10.1111/j.1462-2920.2007.01520.x 18177373

[B10] Clermont O. Olier M. Hoede C. Diancourt L. Brisse S. Keroudean M. (2011b). Animal and human pathogenic *Escherichia coli* strains share common genetic backgrounds. *Infect. Genet. Evol.* 11 654–662. 10.1016/j.meegid.2011.02.005 21324381

[B11] Eisen M. B. Spellman P. T. Brown P. O. Botstein D. (1998). Cluster analysis and display of genome-wide expression patterns. *Proc. Natl. Acad. Sci. U.S.A.* 95 14863–14868. 10.1073/pnas.95.25.14863 9843981 PMC24541

[B12] Gordon D. M. Clermont O. Tolley H. Denamur E. (2008). Assigning *Escherichia coli* strains to phylogenetic groups: multi-locus sequence typing versus the PCR triplex method. *Environ. Microbiol.* 10 2484–2496. 10.1111/j.1462-2920.2008.01669.x 18518895

[B13] Herzer P. J. Inouye S. Inouye M. Whittam T. S. (1990). Phylogenetic distribution of branched RNA-linked multicopy single-stranded DNA among natural isolates of *Escherichia coli*. *J. Bacteriol.* 172 6175–6181. 10.1128/jb.172.11.6175-6181.1990 1699928 PMC526797

[B14] Huberty C. J. (1994). *Applied Discriminant Analysis.* New York, NY: Wiley.

[B15] Hussein A. H. Ghanem I. A. Eid A. A. Ali M. A. Sherwood J. S. Li G. (2013). Molecular and phenotypic characterization of *Escherichia coli* isolated from broiler chicken flocks in Egypt. *Avian Dis.* 57 602–611. 10.1637/10503-012513-Reg.1 24283125

[B16] Jaureguy F. Landreau L. Passet V. Diancourt L. Frapy E. Guigon G. (2008). Phylogenetic and genomic diversity of human bacteremic *Escherichia coli* strains. *BMC Genomics* 9:560 10.1186/1471-2164-9-560 PMC2639426 19036134

[B17] Johnson J. R. Oswald E. O’Bryan T. T. Kuskowski M. A. Spanjaard L. (2002). Phylogenetic distribution of virulence-associated genes among *Escherichia coli* isolates associated with neonatal bacterial meningitis in the Netherlands. *J. Infect. Dis.* 185 774–784. 10.1086/339343 11920295

[B18] Johnson J. R. Stell A. L. (2000). Extended virulence genotypes of *Escherichia coli* strains from patients with urosepsis in relation to phylogeny and host compromise. *J. Infect. Dis.* 181 261–272. 10.1086/315217 10608775

[B19] Johnson T. J. Johnson S. J. Nolan L. K. (2006a). Complete DNA sequence of a ColBM plasmid from avian pathogenic *Escherichia coli* suggests that it evolved from closely related ColV virulence plasmids. *J. Bacteriol.* 188 5975–5983. 16885466 10.1128/JB.00204-06 PMC1540072

[B20] Johnson T. J. Kariyawasam S. Wannemuehler Y. Mangiamele P. Johnson S. J. Doetkott C. (2007a). The genome sequence of avian pathogenic *Escherichia coli* strain O1:K1:H7 shares strong similarities with human extraintestinal pathogenic *E. coli* genomes. *J. Bacteriol.* 189 3228–3236. 17293413 10.1128/JB.01726-06 PMC1855855

[B21] Johnson T. J. Logue C. M. Johnson J. R. Kuskowski M. A. Sherwood J. S. Barnes H. J. (2012a). Associations between multidrug resistance, plasmid content, and virulence potential among extraintestinal pathogenic and commensal *Escherichia coli* from humans and poultry. *Foodborne Pathog. Dis.* 9 37–46. 10.1089/fpd.2011.0961 21988401 PMC3250628

[B22] Johnson T. J. Logue C. M. Wannemuehler Y. Kariyawasam S. Doetkott C. DebRoy C. (2009). Examination of the source and extended virulence genotypes of *Escherichia coli* contaminating retail poultry meat. *Foodborne Pathog. Dis.* 6 657–667. 10.1089/fpd.2009.0266 19580453 PMC3145168

[B23] Johnson T. J. Siek K. E. Johnson S. J. Nolan L. K. (2006b). DNA sequence of a ColV plasmid and prevalence of selected plasmid-encoded virulence genes among avian *Escherichia coli* strains. *J. Bacteriol.* 188 745–758. 16385064 10.1128/JB.188.2.745-758.2006 PMC1347294

[B24] Johnson T. J. Wannemuehler Y. Doetkott C. Johnson S. J. Rosenberger S. C. Nolan L. K. (2008a). Identification of minimal predictors of avian pathogenic *Escherichia coli* virulence for use as a rapid diagnostic tool. *J. Clin. Microbiol.* 46 3987–3996. 10.1128/JCM.00816-08 18842938 PMC2593276

[B25] Johnson T. J. Wannemuehler Y. M. Johnson S. J. Logue C. M. White D. G. Doetkott C. (2007b). Plasmid replicon typing of commensal and pathogenic *Escherichia coli* isolates. *Appl. Environ. Microbiol.* 73 1976–1983. 17277222 10.1128/AEM.02171-06 PMC1828809

[B26] Johnson T. J. Wannemuehler Y. Johnson S. J. Stell A. L. Doetkott C. Johnson J. R. (2008b). Comparison of extraintestinal pathogenic *Escherichia coli* strains from human and avian sources reveals a mixed subset representing potential zoonotic pathogens. *Appl. Environ. Microbiol.* 74 7043–7050. 10.1128/AEM.01395-08 18820066 PMC2583479

[B27] Johnson T. J. Wannemuehler Y. Kariyawasam S. Johnson J. R. Logue C. M. Nolan L. K. (2012b). Prevalence of avian-pathogenic *Escherichia coli* strain O1 genomic islands among extraintestinal and commensal *E. coli* isolates. *J. Bacteriol.* 194 2846–2853. 10.1128/JB.06375-11 22467781 PMC3370642

[B28] Johnson T. J. Wannemeuhler Y. M. Scaccianoce J. A. Johnson S. J. Nolan L. K. (2006c). Complete DNA sequence, comparative genomics, and prevalence of an IncHI2 plasmid occurring among extraintestinal pathogenic *Escherichia coli* isolates. *Antimicrob. Agents Chemother.* 50 3929–3933. 16940062 10.1128/AAC.00569-06 PMC1635206

[B29] Lecointre G. Rachdi L. Darlu P. Denamur E. (1998). *Escherichia coli* molecular phylogeny using the incongruence length difference test. *Mol. Biol. Evol.* 15 1685–1695. 10.1093/oxfordjournals.molbev.a025895 9866203

[B30] Lescat M. Clermont O. Woerther P. L. Glodt J. Dion S. Skurnik D. (2013). Commensal *Escherichia coli* strains in Guiana reveal a high genetic diversity with host-dependant population structure. *Environ. Microbiol. Rep.* 5 49–57. 10.1111/j.1758-2229.2012.00374.x 23757130

[B31] Logue C. M. Doetkott C. Mangiamele P. Wannemuehler Y. M. Johnson T. J. Tivendale K. A. (2012). Genotypic and phenotypic traits that distinguish neonatal meningitis *Escherichia coli* from fecal *E. coli* isolates of healthy human hosts. *Appl. Environ. Microbiol.* 78 5824–5830. 10.1128/AEM.07869-11 22706051 PMC3406136

[B32] McLachlan G. J. (1992). *Discriminant Analysis and Statistical Pattern Recognition.* New York, NY: Wiley 10.1002/0471725293

[B33] Nicholson B. A. Wannemuehler Y. M. Logue C. M. Li G. Nolan L. K. (2016a). Complete genome sequence of the neonatal meningitis-causing *Escherichia coli* strain NMEC O18. *Genome Announc.* 4:e01239-16 10.1128/genomeA.01239-16 PMC5095484 27811114

[B34] Nicholson B. A. West A. C. Mangiamele P. Barbieri N. Wannemuehler Y. Nolan L. K. (2016b). Genetic characterization of ExPEC-like virulence plasmids among a subset of NMEC. *PLoS ONE* 11:e0147757 10.1371/journal.pone.0147757 PMC4723317 26800268

[B35] Nolan L. K. Barnes H. J. Vaillancourt J.-P. Abdul-Aziz T. Logue C. M. (2013). *Colibacillosis.* Hoboken, NJ: Wiley-Blackwell.

[B36] Obata-Yasuoka M. Ba-Thein W. Tsukamoto T. Yoshikawa H. Hayashi H. (2002). Vaginal *Escherichia coli* share common virulence factor profiles, serotypes and phylogeny with other extraintestinal *E. coli*. *Microbiology* 148(Pt 9) 2745–2752. 10.1099/00221287-148-9-2745 12213921

[B37] Picard B. Garcia J. S. Gouriou S. Duriez P. Brahimi N. Bingen E. (1999). The link between phylogeny and virulence in *Escherichia coli* extraintestinal infection. *Infect. Immun.* 67 546–553. 9916057 10.1128/iai.67.2.546-553.1999 PMC96353

[B38] Rodriguez-Siek K. E. Giddings C. W. Doetkott C. Johnson T. J. Fakhr M. K. Nolan L. K. (2005a). Comparison of *Escherichia coli* isolates implicated in human urinary tract infection and avian colibacillosis. *Microbiology* 151(Pt 6) 2097–2110. 15942016 10.1099/mic.0.27499-0

[B39] Rodriguez-Siek K. E. Giddings C. W. Doetkott C. Johnson T. J. Nolan L. K. (2005b). Characterizing the APEC pathotype. *Vet. Res.* 36 241–256. 15720976 10.1051/vetres:2004057

[B40] Selander R. K. Caugant D. A. Whittam T. S. (1987). “Genetic structure and variation in natural populations of *Escherichia coli*,” in *Escherichia coli and Salmonella Typhimurium: Cellular and Molecular Biology* eds Neidhardt F. C. Ingraham J. L. Low K. B. Mahasanik B. Schaechter M. Umbarger H. E. (Washington, DC: ASM Press) 1625–1648.

[B41] Snedecor G. Cochran W. A. (1989). *Statistical Methods.* Ames, IA: Iowa State University Press.

[B42] Tivendale K. A. Logue C. M. Kariyawasam S. Jordan D. Hussein A. Li G. (2010). Avian-pathogenic *Escherichia coli* strains are similar to neonatal meningitis *E. coli* strains and are able to cause meningitis in the rat model of human disease. *Infect. Immun.* 78 3412–3419. 10.1128/IAI.00347-10 20515929 PMC2916289

[B43] Tzipori S. Gunzer F. Donnenberg M. S. de Montigny L. Kaper J. B. Donohue-Rolfe A. (1995). The role of the eaeA gene in diarrhea and neurological complications in a gnotobiotic piglet model of enterohemorrhagic *Escherichia coli* infection. *Infect. Immun.* 63 3621–3627. 7642299 10.1128/iai.63.9.3621-3627.1995 PMC173502

[B44] Walk S. T. Alm E. W. Gordon D. M. Ram J. L. Toranzos G. A. Tiedje J. M. (2009). Cryptic lineages of the genus *Escherichia*. *Appl. Environ. Microbiol.* 75 6534–6544. 10.1128/AEM.01262-09 19700542 PMC2765150

