# Supplementary material for: Comparative Analysis of Phylogenetic Assignment of Human and Avian ExPEC and Fecal Commensal Escherichia coli Using the (Previous and Revised) Clermont Phylogenetic Typing Methods and its Impact on Avian Pathogenic Escherichia coli (APEC) Classification
Source: Front Microbiol. 2017 Feb 23;8:283. doi: 10.3389/fmicb.2017.00283 (PMC5322314; doi:10.3389/fmicb.2017.00283)
Supplement: Supplementary file 1 [file Table_1.pdf]

Supplementary Table 1. Primers used for the amplification of genes/traits of interest

Gene/Trait	Amplicon Size (bp)	Primer Seq. (5'-3')	Reference/Comments
**<u>Plasmid PAI-Encoded Traits:</u>**			
1. *cvaA*	319	F ATCCGGGCGTTGTCTGACGGGAAAGTTG<br>R ACCAGGGAACAGAGGCACCCGGCGTATT	Col V operon
2. *cvaB3'*	550	F GGCCCGTGCCGCCTCCTATTTTA<br>R TCCCGCACCGGAAGCACCAGTTAT	Col V operon
3. *cvaB5'*	247	F TGGCCACCCGGGCTCTTTCACTGGAGTT<br>R ATGCGGGTCTGCAGGGTTTCCGACTGGA	Col V operon
4. *cvaC**	679	F CACACACAAACGGGAGCTGTT<br>R CTTCCCGCAGCATAGTTCCAT	(Johnson and Stell, 2000)
5. *iroN*†	667	F AAGTCAAAGCAGGGGTTGCCCG<br>R GACGCCGACATTAAGACGCAG	(Rodriguez-Siek et al., 2005)
6. *aerJ*	302	F GGCTGGACATCATGGGAACTGG<br>R CGTCGGGAACGGGTAGAATCG	(Johnson and Stell, 2000)
7. *sitA*†	608	F AGGGGGCACAACTGATTCTCG<br>R TACCGGGCCGTTTTCTGTGC	(Runyen-Janecky et al., 2003)
8. *traT**	290	F GGTGTGGTGCGATGAGCACAG<br>R CACGGTTCAGCCATCCCTGAG	(Johnson and Stell, 2000)
9. *tsh*‡	420	F GGGAAATGACCTGAATGCTGG<br>R CCGCTCATCAGTCAGTACCAC	(Maurer et al., 1998)
10. *iss*	323	F CAGCAACCCGAACCACTTGATG<br>R AGCATTGCCAGAGCGGCAGAA	(Johnson et al., 2008)
11. *ompTp*	496	F TCATCCCGGAAGCCTCCCTCACTACTAT<br>R TAGCGTTTGCTGCACTGGCTTCTGATAC	plasmid encoded OMP
12. *hlyF*	599	F GGCGATTTAGGCATTCCGATACTC<br>R ACGGGGTCGCTAGTTAAGGAG	Hemolysin F
13. *etsA*	450	F CAACTGGGCGGGAACGAAATCAGGA<br>R TCAGTTCCGCGCTGGCAACAACCTAC	*E. coli* transport system
14. *etsB*	537	F CAGCAGCGCTTCGGACAAAATCTCCT<br>R TTCCCCACCACTCTCCGTTCTCAAAC	*E. coli* transport system
15. *eitA*	284	F ACGCCGGGTTAATAGTTGGGAGATAG<br>R ATCGATAGCGTCAGCCCGGAAGTTAG	*E. coli* iron transport
16. *eitB*	380	F TGATGCCCCGCCAAACTCAAGA<br>R ATGCGCCGGCCTGACATAAGTGCTAA	*E. coli* iron transport

17. *colB*	430	F ACAAGACAGCACCAGTTATGGGTATT<br>R GTTGTTGGTTTTGTTGGCGTAGTTAT	Colicin B immunity
18. *colM*	498	F CAGCGCCATTACCCCATAAATAGTGA<br>R GGTTCGTTCGCCGGTGTAAGCGTTAG	Colicin M activity
**<u>Chromosomally Encoded Traits:</u>**			
**<u>Adhesins:</u>**			
19. *bmaE*	507	F ATGGCGCTAACTTGCCATGCTG<br>R AGGGGGACATATAGCCCCCTTC	(Johnson and Stell, 2000)
20. *fimH*	508	F TCGAGAACGGATAAGCCGTGG<br>R GCAGTCACCTGCCCTCCGGTA	(Johnson and Stell, 2000)
21. *focG*	364	F CAGCACAGGCAGTGGATACGA<br>R GAATGTCGCCTGCCCATTGCT	(Johnson and Stell, 2000)
22. *gafD*	952	F TGTTGGACCGTCTCAGGGCTC<br>R TCCCGGAACTCGCTGTTACT	(Johnson and Stell, 2000)
23. *papA*	717	F ATGGCAGTGGTGTTTTGGTG<br>R CGTCCCACCATACGTGCTCTTC	(Johnson and Stell, 2000)
24. *papC*	205	F GTGGCAGTATGAGTAATGACCGTTA<br>R ATATCCTTTCTGCAGGGATGCAATA	(Johnson and Stell, 2000)
25. *papEF*	326	F GCAACAGCAACGCTGGTTGCATCAT<br>R AGAGAGAGCCACTCTTATACGGACA	(Johnson and Stell, 2000)
26. *papG* allele I§	461	F TCGTGCTCAGGTCCGGAATTT<br>R TGGCATCCCCCAACATTATCG	(Johnson and Stell, 2000)
27. *papG* allele I'§	479	F CTACTATAGTTCATGCTCAGGTC<br>R CCTGCATCCTCCACCATTATCGA	(Johnson and Stell, 2000)
28. *papG* allele II§	190	F GGGATGAGCGGGCCTTTGAT<br>R CGGGCCCCCAAGTAACTCG	(Johnson and Stell, 2000)
29. *papG* allele III§	258	F GGCCTGCAATGGATTTACCTGG<br>R CCACCAAATGACCATGCCAGAC	(Johnson and Stell, 2000)
30. *papG1**	1140	F CTGTAATTACGGAAGTGATTTCTG<br>R TTCCAGAAATAGCTCATGTAACCCG	
31. *papG2/3*	1070	R ACTATCCGGCTCCGGATAAACCAT	
32. *sfaS*	244	F GTGGATACGACGATTACTGTG<br>R CCGCCAGCATTCCCTGTATTC	(Johnson and Stell, 2000)
33. *sfa-foc*	410	F CTCCGGAGAACTGGGTGCATCTTAC<br>R CGGAGGAGTAATTACAAACCTGGCA	(Johnson and Stell, 2000)
34. *eaeH*	605	F AAAAGCCGCTGGTGCTGTCTCTGC<br>R AAGCGGCCCGGCAACAAACTTC	EAE Locus

35. *aufA*	515	F CCGCGCTGAAATCATGGGTCGTAG<br>R TGGGGCGGATACTGACTGGAGCAC	Auf fimbra
36. *aufC*	298	F CGCCAACGCCCGGTAATCCTC<br>R GCCGCCGGGACCGTTTGAGAT	Auf fimbra
37. *aufE*	269	F CAAGCCCCTCTACCGAGCCATCTT<br>R TCATGCCGGAGTGGTTCGTATTGA	Auf fimbra
38. *aufG*	453	F TCGCCCGCTGATCGCGTGTAAA<br>R CCGCCACCATTAGCCGTCTTTGTG	Auf fimbra
39. *iha*	829	F CTGGCGGAGGCTCTGAGATCA<br>R TCCTTAAGCTCCCGCGGCTGA	UPEC Island
**<u>Iron-Related:</u>**			
40. *feoB*	470	F AATTGGCGTGCATGAAGATAACTG<br>R AGCTGGCGACCTGATAGAACAATG	(Runyen-Janecky et al., 2003)
41. *fyuA*	787	F TGATTAACCCCGCGACGGGAA<br>R CGCAGTAGGCACGATGTTGTA	(Johnson and Stell, 2000)
42. *fyuA1*	390	F AACCCCGCCGTCACCCTGTATGTC<br>R GTCCGGCGCCAGACGCAGTTT	
43. *ireA*	254	F GATGACTCAGCCACGGGTAA<br>R CCAGGACTCACCTCACGAAT	(Rodriguez-Siek et al., 2005)
44. *irp*-2	287	F AAGGATTCGCTGTTACCGGAC<br>R TCGTCGGGCAGCGTTTCTTCT	(Janben et al., 2001)
**<u>Protectins:</u>**			
45. *kpsMT K1*	153	F TAGCAAACGTTCTATTGGTGC<br>R CATCCAGACGATAAGCATGAGCA	(Johnson and Stell, 2000)
46. *kpsMT II*	272	F GCGCATTTGCTGATACTGTTG<br>R CATCCAGACGATAAGCATGAGCA	(Johnson and Stell, 2000)
47. *kpsMT III*	392	F TCCTCTTGCTACTATTCCCCCT<br>R AGGCGTATCCATCCCTCCTAAC	(Johnson and Stell, 2000)
**<u>Toxins:</u>**			
48. *cdtB*	430	F GAAAATAAATGGAACACACATGTCCG<br>F’ GAAAGTAAATGGAATATAAATGTCCG<br>R AAATCTCCTGCAATCATCCAGTTA<br>R’ AAATCACCAAGAATCATCCAGTTA	(Johnson and Stell, 2000)
49. *cnf*-1	1105	F ATCTTATACTGGATGGGATCATCTTGG<br>R GCAGAACGACGTTCTTCATAAGTATC	(Johnson and Stell, 2000)
50. *hlyD*	904	F CTCCGGTACGTGAAAAGGAC<br>R GCCCTGATTACTGAAGCCTG	(Rodriguez-Siek et al., 2005)

51. *vat*	370	F GAGGCCGGCGACCAGTGGTTCTCCATTA<br>R TGAACCGGCACCCTGCGTGACAGAGTTT	Vacuolating autotransporter
invasins			
52. *ibeA*	171	F AGGCAGGTGTGCGCCGCGTAC<br>R TGGTGCTCCGGCAAACCATGC	(Johnson and Stell, 2000)
53. *gimB*	619	F AAAAGCCGCCCAGGAATCAGAAGAACAA<br>R TTACCCACAAAAGGCCGGGGAGTGATAA	ExPEC Gim B island
**Miscellaneous:**			
54. *rfc*	788	F ATCCATCAGGAGGGGACTGGA<br>R AACCATACCAACCAATGCGAG	O antigen polymerase
55. *adhE*	411	F GTCGGCGAGGGCGTCACCAGCCTGAAGC<br>R GGCCGCGCCCTGAATCACCGCCAGACC	(Johnson et al., 2007a)
56. *fliC* (H7)	547	F ACGATGCAGGCAACTTGACG<br>R GGGTTGGTCGTTGCAGAACC	(Rodriguez-Siek et al., 2005)
57. *ompTc*	559	F ATCTAGCCGAAGAAGGAGGC<br>R CCCGGGTCATAGTGTTCATC	(Rodriguez-Siek et al., 2005)
58. *malX PAI*	925	F GGACATCCTGTTACAGCGCGCA<br>R TCGCCACCAATCACAGCCGAAC	(Johnson and Stell, 2000)
59. O2ColV39	437	F TACTGCCCGGTGTGATATTTCCAACAAA<br>R TAAGATTGACAATTGCAGGACCGTAACC	ColV hypothetical gene
60. 1024UI	598	F GGCTTTCCCGCCTTCTTTTACCACTACG<br>R GGACGGCGACGTTGTGTTATTCGGTAAT	Inc FIB recombinase/integrase
61. 1051UI	664	F CACGCCGTTACTGGTCGCGGAAAAAT<br>R AACCCACGGCCTCTATTGGCGAAGAACT	ColV gene
62. *parB*	534	F TCGTGGCCGAGTTCTTGGCAACAGC<br>R GCGGCCTGAAACGCACGAGTCACTTT	Inc FIB Plasmid partitioning
63. *umuC*	474	F CCGCCGTACGGAAAAACTGCTGTCACTG<br>R ACGGCAGCGGCAATGATGTCCTGTGTAT	UV protection gene
64. *afa*	594	F GGCAGAGGGCCGGCAACAGGC<br>R CCCGTAACGCGCCAGCATCTC	Afimbrial adhesin Afa
65. *groEL*	318	F CGCCGGCATGAACCCGATGGACCTCA<br>R TCGGCCTGCATCGACTGCGGGTTGTTG	Chaperone
66. *iseC12*	404	F CGCGGCCACGTAAACCGAAAGATAAA<br>R GCGCGGGTGCACAGCAACCTC	Transposase

Resistance genes

67. *blaTEM*	558	F ATGTGCGCGGAACCCCTATTTGTTTA<br>R AAAAAGCGGTTAGCTCCTTCGGTCCT	Ampicillin resistance
68. *aac3 VIa*	502	F GGCACCCGCGACGCCCTGGTCCAAAAG<br>R GGGCCCGGCGCCGATCGACAGGATTT	Gentamicin resistance
69. *aac3 VIb*	302	F GGGCAAGCGCCGCGTCACTTATT<br>R CGCGGCGTTGTTTCGGCTTCA	Gentamicin resistance
70. *tetA*	372	F CGGGGCGACTGGGGCGGTAGC<br>R CAAAGCGCGGCCGGCACCTGT	Tetracycline resistance
71. *tetB*	446	F AACGCGTGAAGTGGTTCGGTTGGT<br>R TTCGCCCCATTTAGTGGCTATTCTTC	Tetracycline resistance
72. *aph3IA*	378	F TCGGGCAATCAGGTGCGACAATCTA<br>R TGCCAGCGCATCAACAATATTTTCACC	Gentamicin resistance
73. *terD*	231	F CCACTGCGCGGAATTTCCACTCACCAT<br>R ACGCCGTCCCGTCTGATGTTGACAAG	Tellurite resistance
74. *terF*	428	F CCGACAAACTTCCAGAAGATGGGGTAGT<br>R GAGGCAGCGGTTGCATTTGTACTTGACG	Tellurite resistance
75. *terX*	576	F ATGCGCCGCCTGCCTGTTTACCTTGTTA<br>R CGCGCTTGTGCTGCCGGAAGACA	Tellurite resistance
76. *terY3*	302	F CCTGGGGCCGTCAGCGGACCTG<br>R TCCTTGCTGGTGGCCGTTCATACTTCAT	Tellurite resistance
77. *pcoA*	507	F ATCCGGAAGGTCAGCACCGTCCATAGAC<br>R GACCTCGCGGATGTCAGTGGCTACACCT	Copper resistance
78. *pcoD*	502	F GGCGCCCAGAATGATAATCGCAACA<br>R GGGCGTGGCGCTGGCTACACTT	Copper resistance
79. *pcoE*	385	F GTGGGGCAGCTTTTGCTCAGTCCAGTGA<br>R CGAAGCTTTCTTGCCTGCGTCTGATGTG	Copper resistance
80. *dfr1*	328	F ATCGGGAATGGCCCTGATA<br>R CTTCCGGCTCGATGTCTATTGTAG	Trimethoprim resistance
81. *dfr7*	214	F TCTTTAAAGCGCTCACATATAATCAGTG<br>R ATTTGACCGCCACCAGAGACA	Trimethoprim resistance
82. *dfr17*	243	F ATATCCCGTGGTCAGTAAAAGGTG<br>R GACCCCCGCCAGAGACATA	Trimethoprim resistance
83. *arsC*	153	F CCAGCCTGCGGCACCTCGCGTAATAC<br>R ACGCAGCAGCGCTCGTACTGAAATACCC	Arsenic resistance
84. *silE*	364	F TCGGCCTGGGCCACTGAAACCGTGAATA	Silver

		R GGCGGTGCGCTTCGGCCATAGCCTGATG	resistance
85. *silP*	603	F ACACCCCGGCCTGGGCTCCTT<br>R TGCGGGCACGGGAACAAACCTC	Silver<br>resistance
86. *sul1*	462	F CGCCGCTCTTAGACGCCCTGTCC<br>R CAACGGTGGCGCCCAAGAAGGAT	Sulfa resistance
87. *merA*	250	F GATCCGCGCCGCCCATATCGCCCATCTG<br>R CACGCGCTCGCCGCCGTCGTTGAGTTG	Mercury<br>resistance
88. *intI1*	545	F CACTCCGGCACCGCCAACTTTC<br>R GAACGGGCATGCGGATCAGTGAG	Integrase
89. *aadA*	365	F TAACGGCGCAGTGGCGGTTTTCA<br>R AAGCTCGCCGCGTTGTTTCATCAAG	Aminoglycoside<br>resistance
90. *qacE delta*	250	F TCGGCCTCCGCAGCGACTTCC<br>F CTTGCCCCTTCCGCCGTTGTCTAAT	Quaternary<br>ammonium compounds
91. *qnr*	440	F TCGCCGCTGCCGCTTTTATCAGT<br>R GCCAACAGTCGCGGGAGAAGGTG	qnr plasmid<br>fluoroquinolone resistance
**<u>Plasmid-Replicon Types:</u>**			
92. B/O	159	F GCGGTCCGGAAAGCCAGAAAAC<br>R TCTGCGTTCCGCCAAGTTCGA	(Johnson et al., 2007b)
93. FIC	262	F GTGAACTGGCAGATGAGGAAGG<br>R TTCTCCTCGTCGCCAAACTAGAT	(Johnson et al., 2007b)
94. A/C	465	F GAGAACCAAAGACAAAGACCTGGA<br>R ACGACAAACCTGAATTGCCTCCTT	(Johnson et al., 2007b)
95. P	534	F CTATGGCCCTGCAAACGCGCCAGAAA<br>R TCACGCGCCAGGGCGCAGCC	(Johnson et al., 2007b)
96. T	750	F TTGGCCTGTTTGTGCCTAAACCAT<br>R CGTTGATTACACTTAGCTTTGGAC	(Johnson et al., 2007b)
97. K/B	160	F GCGGTCCGGAAAGCCAGAAAAC<br>R TCTTTCACGAGCCCGCCAAA	(Johnson et al., 2007b)
98. W	242	F CCTAAGAACAACAAAGCCCCCG<br>R GGTGCGCGGCATAGAACCGT	(Johnson et al., 2007b)
99. FIIA	270	F CTGTCGTAAGCTGATGGC<br>R CTCTGCCACAAACTTCAGC	(Johnson et al., 2007b)
100. FIA	462	F CCATGCTGGTTCTAGAGAAGGTG<br>R GTATATCCTTACTGGCTTCCGCAG	(Johnson et al., 2007b)
101. FIB	702	F GGAGTTCTGACACACGATTTTCTG<br>R CTCCCGTCGCTTCAGGGCATT	(Johnson et al., 2007b)

102.Y	765	F AATTCAAACAACACTGTGCAGCCTG<br>R GCGAGAATGGACGATTACAAAACTTT	(Johnson et al., 2007b)
103.I1	139	F CGAAAGCCGGACGGCAGAA<br>R TCGTCGTTCCGCCAAGTTCGT	(Johnson et al., 2007b)
104.X	376	F AACCTTAGAGGCTATTTAAGTTGCTGAT<br>R TGAGAGTCAATTTTTATCTCATGTTTTAGC	(Johnson et al., 2007b)
105.HI1	471	F GGAGCGATGGATTACTTCAGTAC<br>R TGCCGTTTCACCTCGTGAGTA	(Johnson et al., 2007b)
106.N	559	F GTCTAACGAGCTTACCGAAG<br>R GTTTCAACTCTGCCAAGTTC	(Johnson et al., 2007b)
107.HIII	644	F TTTCTCCTGAGTCACCTGTTAACAC<br>R GGCTCACTACCGTTGTCATCCT	(Johnson et al., 2007b)
108.L/M	785	F GGATGAAAACTATCAGCATCTGAAG<br>R CTGCAGGGGCGATTCTTTAGG	(Johnson et al., 2007b)
**APEC O1 Genomic Islands of Unknown Function:**			
109.PAI 15-1	392	F TACCGGCGTGATGGCCACCTACA<br>R AAACAACGCCGGTTCTGCCTACCAG	APEC O1 genomic island[a]
110.PAI 15-2	313	F AAGCTATCCCGACTTGCTCTCGCTGTAA<br>R AAGGCGCTGGCACTGCAACAACTAC	APEC O1 genomic island[a]
111.PAI 15-3	468	F AGCGGTCACAGTGGCTTTCATAGGTG<br>R GCCCCGCTTTTCCAGGCACAC	APEC O1 genomic island[a]
112.PAI 15-4	533	F CAGGGATTCGCTTGCCTCTTCTGTGTA<br>R CGACCGCCGGGATTTTAACCATAC	APEC O1 genomic island[a]
113.PAI 15-5	215	F GGTGGCGAGATAACCGACGTAGTGGT<br>R CCCCTGGCGAATGGAAAACAGAGAT	APEC O1 genomic island[a]
114.PAI 15-6	366	F CCGGCTCAGGAGAGTCTGTTAATACACT<br>R TGATAGCCCGCTCCAGATTTTCTTTAG	APEC O1 genomic island[a]
115.PAI 15-7	266	F TTGACCTGTACCCGCATAAACACTGGTT<br>R TGCGAGAGGTGCTTAACGCGATTTTC	APEC O1 genomic island[a]
116.PAI 16-1	358	F CCCCACAGGCCGCTCCACAG<br>R TGCCCGATAGGCGAAGATGGGTTAT	APEC O1 genomic island[a]
117.PAI 16-2	446	F CTGGCCCAACCCCACCGTTATCTT<br>R ATGGCCGGGTACGGGTGAAGTTTC	APEC O1 genomic island[a]
118.PAI 16-3		F ATCGGCACCAGAAGTACCGGATTTGA<br>R GTCGGCAATATCTACGTGCAGGGACAC	APEC O1 genomic island[a]

119. PAI 16-4	310	F GGAGGCGGCGGGTTTTGACAA<br>R GTATTCCGCCATCCGCCCATCC	APEC O1<br>genomic island[a]
120. PAI 16-5	529	F AGCGGCGTCCTGCTGCTTACTCTG<br>R CCGGCACCGGGGAGAAAATGA	APEC O1<br>genomic island[a]
121. PAI 16-6	573	F GTCAGCCGCTGGACCCCCTATGAT<br>R TATCGCCGCTTCCCTCGCCTTATC	APEC O1<br>genomic island[a]
122. PAI 16-7	250	F CTGAGCCGTGCGCAGAATGTCAAC<br>R ACTCTTCCGCCTGCCCTGGACTGT	APEC O1<br>genomic island[a]
123. PAI 8-1	410	F AGCCGGGCGGTCATGCTGAGTTATGGATTA<br>R CCAGCGAGGAACGGCAAGATGCTTACCAA	APEC O1<br>genomic island[a]
124. PAI 8-2	355	F ATCGCCGCGCCTCGGAGTCCTGT<br>R CTCCACGCGCCAGCGCCTCAAG	APEC O1<br>genomic island[a]
125. PAI 8-3	463	F CGAGGTCGGCGTGGCTGGCTTCCT<br>R TCCCGCCCGGTCTGGATTGGTTACGAC	APEC O1<br>genomic island[a]
126. PAI 8-4	277	F ACCGCCCTGACCGAAAAGGTGAAATCCATT<br>R CTTTACTGCGGCGCTGCTGGGTCAGACTTT	APEC O1<br>genomic island[a]
127. PAI 8-5	317	F GCCGCAAATCCGGGACTGGCTGAACT<br>R ATACCGCACACCGCAGGCAGTGACAGG	APEC O1<br>genomic island[a]
128. PAI 8-6	515	F CGCGCAGTTTTGACGGGCGAATGAC<br>R GACCCGGCGACGCTTAAGCCCTCAAAC	APEC O1<br>genomic island[a]
129. PAI 8-7	227	F GGCCGCTGTGGGAATTAATACCCGCAATCT<br>R CCGCCGCCGCAATCCCCACAC	APEC O1<br>genomic island[a]
130. PAI 8-8	159	F TATGCGCCAGACGCTGCCACACCAGAC<br>R CCGCCGGTCAGTTCGGGATAAAGCACAC	APEC O1<br>genomic island[a]
131. PAI 13-1	404	F TTTTTGCATTGGTGGCGGAGCGTGGTAT<br>R TATCGGCCAGGCGGGGAGTAAGTTTGTCAT	APEC O1<br>genomic island[a]
132. PAI 13-2	308	F GTCGCGTTATGGCGGCTGAAGTGCTCAAA<br>R GCCCGACGGCTCATAAACAGGCGTATCAA	APEC O1<br>genomic island[a]
133. PAI 13-3	495	F TATGAGGGGCAAGGTGCGACAGCTAACAGC<br>R ACGCCGGGCGGCAGCCTTATCCT	APEC O1<br>genomic island[a]
134. PAI 13-4	437	F ATCCGCCATATTATGCCCCGTCGAGTTGAA<br>R AGAAGTTTAGCGCCGGCATGACGCATCTG	APEC O1<br>genomic island[a]
135. PAI 13-5	236	F GTACCGGGGCCGTCAGCTCCGAATACC<br>R ACGGCGCGGGAAGCACCGAAATACA	APEC O1<br>genomic island[a]

136.PAI 13-6	364	F TGTCCCCGCAGGTTCTGGTTCAAAACTCAT<br>R GAGCCATCCGGGAGCCACCAATCTCATTAT	APEC O1<br>genomic island[a]
137.PAI 13-7	532	F TGGCTGCGGCGCTAAGAAATCTTGAACAAC<br>R GCCGGGCCAATCCCACCGAACATA	APEC O1<br>genomic island[a]
138.PAI 17-1	197	F CGCCGTCGCTGCGGGTGATGGTAA<br>R GTCACTGGCCGGAATTAAAATCCCGCACAT	APEC O1<br>genomic island[a]
139.PAI 17-2	242	F CTGCCGTGCCTTTTCAAGTGCATGACCAGT<br>R CCGGCGAAGCGGAAACACGAATGAGTAA	APEC O1<br>genomic island[a]
140.PAI 17-3	418	F TGCGCGGGTGCACAGCAACCTC<br>R CTGCGCGGCCACGTAAACCGAAAAGATAAA	APEC O1<br>genomic island[a]
141.PAI 17-4	370	F AGTTCGCCACCGCAATGAGTACGCCTTCA<br>R GGATTATTTCCCGGCCTCGGCCATTCATTT	APEC O1<br>genomic island[a]
142.PAI 17-5	322	F CGCGCCCTGAAAACCGGCCATAACC<br>R GCGCCAGTGTGGACGGGGTGGAGA	APEC O1<br>genomic island[a]
143.PAI 17-6	443	F AGCGCCCTTAACTCTGCCTGGCTGAATGAG<br>R AGCCAGCCGTTTATTTCCCGAATCCTGTCC	APEC O1<br>genomic island[a]
144.PAI 17-7	285	F CGCCGCGAGGCAATTTGCGTAGTTCAC<br>R ATGCGCTCAAAGAGGATGGCGACGAAAGAG	APEC O1<br>genomic island[a]
145.PAI 17-8	495	F AGGAAACACAACGCGTGGCTGACGGGTAAG<br>R TCGCGGGGAGGTGCTTTGATGTCCTGTT	APEC O1<br>genomic island[a]
146.PAI 18-1_	164	F GGCCCGCCGCAGACGCAGCATA<br>R ACCGGCACACCTCCCGCAGTGAACG	APEC O1<br>genomic island[a]
147.PAI 18-2_	207	F GGGGGATAGCACGAAAACAAACCTCA<br>R ATCGGGCAAGATTCAGTAGCGGAAGC	APEC O1<br>genomic island[a]
148.PAI 18-3_	391	F GTCACGGGAATGGCTTAATACACAGG<br>R GAGACCGCCTTTATTCCTTTCTGAGG	APEC O1<br>genomic island[a]
149.PAI 18-4_	561	F ATCAGCGCGGCGACTTTACGGTTAGT<br>R CCTGCGGCAGATTATCTCCCAGTGTG	APEC O1<br>genomic island[a]
150.PAI 18-5_	335	F TTCCGCCACGCACGGCAGGTTGTT<br>R CACATCCGCGCTCGCGCAAAGTGTC	APEC O1<br>genomic island[a]
151.PAI 18-6_	681	F TGAATGCCCGTATGACAAAGTAGGA<br>R GTTGCTTTGCGCTTGTCTTTTAGTCC	APEC O1<br>genomic island[a]
152.PAI 18-7_	456	F CGCCGGTGCGCCCAACTG<br>R GCGATTTCCGCCGCCGTCTT	APEC O1<br>genomic island[a]
153.PAI 18-8_	501	F TGGGCCCGGGCAACCACAA<br>R TGAGGGGGCTGCCAGTGAAAATGAC	APEC O1<br>genomic island[a]

154.PAI 18-9_	260	F GCTACCGCGGAGATGGGGGATTAT	APEC O1
		R CACGCGTTGCGGGTAGGTTTCA	genomic island[a]
155.PAI 19-1	404	F CCTGGGGCAGTGCGAGCAACTTG	APEC O1
		R CGGCGCACATAAATACCCTGGCTACAC	genomic island[a]
156.PAI 19-2	445	F ATATGGGCGAATCGGAAGTGGCAGTT	APEC O1
		R AGGCCGATGAATGCGCAAACCAAC	genomic island[a]
157.PAI 19-3	503	F GGGAGCGCGTGCGGATGGTT	APEC O1
		R ATTGCCGGCTCGTCCTTCCTTTTGA	genomic island[a]
158.PAI 19-4	338	F GATTGCCGAATGTGCCGAGCGTAAA	APEC O1
		R GTGCGCCGACGGCTAATTCTTCAATC	genomic island[a]
159.PAI 19-5	556	F GGGGGCGGATGAAGAAAATAAAAACTG	APEC O1
		R TGCCCGTCCGCAAACCAACAG	genomic island[a]
160.PAI 19-6	250	F AGGCCGATCTGGTGCGCAATTTTATTC	APEC O1
		R AGCCGATCTACGCCTTTTTCAGCAACAC	genomic island[a]
161.PAI 19-7	595	F GGGTGCAGCGGTGAGAGGAAAACTAACT	APEC O1
		R GCGGGCTAAGGCGTTGTTGACCTG	genomic island[a]
162.PAI 19-8	298	F TTGTCGCCCGCAAAACTAACGCTAAACC	APEC O1
		R AATGCGGCGCTGGCCATGATTACC	genomic island[a]
163.PAI 19-9	211	F AGCCGGGAAATTATCAGCCTGGCACTT	APEC O1
		R CTTCGCCTTCGAGCACTTCCTGGATGT	genomic island[a]
164.20-1	420	F CGCTGTCATGGTTTTGGCAAAGAAATC	APEC BEN2908
		R AATCGCGGGTGTTACTTCCGGATACATC	EPI-I
165.20-2	365	F CACCGTGGTCAATGACTTCCTGGACTG	APEC BEN2908
		R AGGTGCGCCATTTAATGTTGCGATAGGT	EPI-I
166.20-3	496	F GAGCTGGCCCTGGGCATCTGC	APEC BEN2908
		R CGGCGGTAACTATCGGGGTAATGATTTC	EPI-I
167.20-4	299	F TTAATCGGGCCAGGAGGCAGAAGAAGA	APEC BEN2908
		R CGGCCCGGCTTATCTGCTTTTTAGTTAT	EPI-I
168.20-5	558	F CCGGCGGTGAAAACGTTCGAAGAGTAT	APEC BEN2908
		R TGCTATAACCCCGGCCTTTCGTTTCTGT	EPI-I
169.20-6	459	F ATTCTCCGCGGCACTCCTCAATGA	APEC BEN2908
		R CAACCACGCAGGGGAAACGCTTTAT	EPI-I
170.20-7	246	F AATTGCCGGAGCGCTGGTTGAAGA	APEC BEN2908
		R ACGGGGCGTTGCCTGATGATACTGAC	EPI-I

171.20-8	209	F ACCGGGGAAATGTGGTGGACAGAGTC<br>R CCGGCGTCGTGGCGTCAGAAT	APEC BEN2908<br>EPI-I
172.20-9	155	F AAGGGCACAGTAACGCAACCGAACCT<br>R GAATGGCATCCGGCCGTATGTTGTT	APEC BEN2908<br>EPI-I
173.PAI 22-1	352	F TAGCGGCCTACGCGTGCCTGAT<br>R GGGCGATTAAGCCGGGAACCACT	APEC O1<br>genomic island[a]
174.PAI 22-2	397	F ATCGGGCCGACAAAATCAGTCCAC<br>R ATGGCCGACGGTTATTGCCTTAGC	APEC O1<br>genomic island[a]
175.PAI 22-8	234	F GGTATTACGCGGGCCGCATTGTC<br>R CCTTGCTGCAACACGGGAGGAAT	APEC O1<br>genomic island[a]
176.PAI 20-1	268	F AAATTACCGCCCCAGCCAAAGCCCCTAAAG<br>R GCCCCCACTCTGCTGGCTCGTTCTGTAAA	APEC O1<br>genomic island[a]
177.PAI 20-2	377	F GCGGTGGGCGTTCTGCGGATTCC<br>R GGCGCGGGCAGAGTATAGACGCATTGGTT	APEC O1<br>genomic island[a]
178.PAI 20-3	443	F CCGCGAGAGTTAATAAATCTGCCGACACCA<br>R GCTGCGGCGGTAACTTCTCTACAGGGTTTT	APEC O1<br>genomic island[a]
179.PAI 20-4	408	F TCCCTGCGTCGAAATCCCTCTGCTCTGAAC<br>R GCGGCGGCCACTATGGGGGCTACC	APEC O1<br>genomic island[a]
180.PAI 20-5	512	F CCGCCATCCCCATCGCCATCATTTGT<br>R GCGGCAGACCATCAACAGCGCCTACTTC	APEC O1<br>genomic island[a]
181.PAI 20-6	322	F TAGTCCGGCGAATGCCTCAGTGTTGTGCTC<br>R TCCAGAGACGCGCTATCAGCCGCTTATGCT	APEC O1<br>genomic island[a]
182.PAI 20-7	214	F CTGTGTCCGGAGGCGTACTCATGGGTTCTG<br>R AGATTCCCGTGGCGGCATCCGGTTATGTA	APEC O1<br>genomic island[a]
183.PAI 20-8	584	F AGGATCGCGGTTCGCAGGGACCAATGTA<br>R GGCGGGCGAACAGTTCAATGCGAGAAAC	APEC O1<br>genomic island[a]
184.PAI 29-1	379	F CATTTCCCGCGGTGGTATGTTTCTGTTT<br>R GATATCAGCGGTAAAGCGAGCGGTGACT	APEC O1<br>genomic island[a]
185.PAI 29-2	422	F CCGGGAAGCGTTCTGACATGACGATTGT<br>R ACCAGCGCAGTTCGCAGCTCTATCAACC	APEC O1<br>genomic island[a]
186.PAI 29-3	340	F CACCGCATCCCGTATAGAGCAGCCTGAA<br>R TGCCTGACCGGTGACTCCGACTGGTT	APEC O1<br>genomic island[a]
187.PAI 29-4	227	F CACCCCTTTAAAACGAGCAGCATTACTG<br>R AACGCCGCCCTATCAGCAGACAACATTT	APEC O1<br>genomic island[a]
188.PAI 29-5	489	F AATCCCCCGGTCAGCCAAGTGCTAACTG<br>R CGACGGCCGCGAGTGCGTAACC	APEC O1<br>genomic island[a]

189. PAI 29-6	273	F GCGGGATCTGCGTGGTCTTCGTCAGT<br>R CGCCCGCTTCCACCCGCAAGTTA	APEC O1<br>genomic island[a]
190. PAI 29-7	158	F GCGGGCGGCATCGGCATTTTACT<br>R CACGGCGGAAAGTCGTACAGCCACAC	APEC O1<br>genomic island[a]
191. PAI 29-8	527	F GTGATGGCGTGGGTGGCGACTCAGTATC<br>R GCCGCGAGGATTGACACCAGCAAGTAAG	APEC O1<br>genomic island[a]
**Miscellaneous:**			
192. *eaeH*	605	F AAAAGCCGCTGGTGCTGTGTCTCTGC<br>R AAGCGGCCCGGCAACAAACTT	EAE locus
193. *irp2*	560	F CACGGCGCCCCGCCGGTGGTAGTATT<br>R AGGACGGCCAGAACCGCAACAAGT	(Janben et al., 2001)
194. *fimH*	508	F TCGAGAACGGATAAGCCGTGG<br>R GCAGTCACCTGCCCTCCGGTA	(Johnson and Stell, 2000)
195. *ratA*	486	F TCGCCGCACCTGAGGATTCGTC<br>R ACAGCGCCAGCATACCCGGTCTACC	(Baumgart et al., 2007)<br>and this study
196. *fepC*	411	F GCCCGATAATTGCCGTGAAGTGA<br>R TCGCGGCTGTTAATGATGCTGGTC	(Simpson et al., 2006) and<br>this study
197. *usp*	352	F CGATGTCGATGGCCCCCTGATT<br>R TGGTGCGCCGTTGCCGTTAT	(Simpson et al., 2006)
198. *dsbA*	274	F GGGGGTGACGTGGGCAAAGAGC<br>R TAACAAACARCGCCGGAACACCAC	(Lin et al., 2008)
199. *yadH*	884	F CTTTATGGCGTAGCGGATTC<br>R GTTGCGTTTAACTGGGAACC	Inner membrane<br>transport protease
200. *96V*	783	F TGGTAGTGTTTGGGGGAGGAG<br>R GCATTTCCTGCAGACAGGTT	colBM<br>autotransporter
201. *pic*	572	F ACTGGATCTTAAGGCTCAGGAT<br>R GACTTAATGTCACTGTTCAGCG	(Simpson et al., 2006)
202. *1523*	527	F GCTGACACCATCATCACGTC<br>R CGACAGTCTGCGGTAACTGA	colBM<br>autotransporter
203. *96P*	467	F ATGGACCGGGATGACAGATA<br>R TAATCCCCTGACCACGTTTC	colBM<br>autotransporter
204. *Vat*	370	F GAGGCCGGCGACCAGTGGTTCTCCA<br>R TGAACCGGCACCCTGCGTGACAGAG	Vacuolating<br>autotransporter
205. *tsh*	304	F CCGTACACAAATACGACGG<br>R GGATGCCCCTGCAGCGT	(Maurer et al., 1998)
206. *sinH*	250	F TGGCAGTACAGTTCCGATCA	Autotransporter

		R GCCGCTGTCGGTTACAT	
207.*fimD2*	600	F ATTATCCGGCAGCAGAGTGCC<br>R CGACACTTGCAGATGGCACC	Fimbrae
208.*manZ2*	300	F TGTTGAGCAGACGAACCATCAGTAGC<br>R CGATGAAGTGGTATGGCCTACAGC	Mannose permease

§ = *pap*G genotypes were assessed using an allele-specific assay developed by Johnson and Brown (Johnson and Brown, 1996).

[a] described in Johnson et al (Johnson et al., 2012)

REFERENCES

Baumgart, M., Dogan, B., Rishniw, M., Weitzman, G., Bosworth, B., Yantiss, R., et al. (2007). Culture independent analysis of ileal mucosa reveals a selective increase in invasive Escherichia coli of novel phylogeny relative to depletion of Clostridiales in Crohn's disease involving the ileum. *ISME.J.* 1(5), 403-418.

Janben, T., Schwarz, C., Preikschat, P., Voss, M., Philipp, H.C., and Wieler, L.H. (2001). Virulence-associated genes in avian pathogenic Escherichia coli (APEC) isolated from internal organs of poultry having died from colibacillosis. *Int.J.Med.Microbiol.* 291(5), 371-378.

Johnson, J.R., and Brown, J.J. (1996). A novel multiply primed polymerase chain reaction assay for identification of variant papG genes encoding the Gal(alpha 1-4)Gal-binding PapG adhesins of Escherichia coli. *J Infect Dis* 173(4), 920-926.

Johnson, J.R., and Stell, A.L. (2000). Extended virulence genotypes of *Escherichia coli* strains from patients with urosepsis in relation to phylogeny and host compromise. *J. Infect. Dis.* 181(1), 261-272. doi: JID990990 [pii]
10.1086/315217.

Johnson, T.J., Kariyawasam, S., Wannemuehler, Y., Mangiamele, P., Johnson, S.J., Doetkott, C., et al. (2007a). The Genome Sequence of Avian Pathogenic *Escherichia coli* Strain O1:K1:H7 Shares Strong Similarities with Human Extraintestinal Pathogenic *E. coli* Genomes. *Journal of Bacteriology* 189(8), 3228-3236. doi: JB.01726-06 [pii]
10.1128/JB.01726-06.

Johnson, T.J., Wannemuehler, Y., Kariyawasam, S., Johnson, J.R., Logue, C.M., and Nolan, L.K. (2012). Prevalence of avian-pathogenic Escherichia coli strain O1 genomic islands among extraintestinal and commensal E. coli isolates. *J Bacteriol* 194(11), 2846-2853. doi: 10.1128/JB.06375-11.

Johnson, T.J., Wannemuehler, Y.M., Johnson, S.J., Logue, C.M., White, D.G., Doetkott, C., et al. (2007b). Plasmid Replicon Typing of Commensal and Pathogenic *Escherichia coli* Isolates. *Applied and Environmental Microbiology* 73(6), 1976-1983. doi: AEM.02171-06 [pii]
10.1128/AEM.02171-06.

Johnson, T.J., Wannemuehler, Y.M., and Nolan, L.K. (2008). Evolution of the *iss* gene in *Escherichia coli*. *Appl. Environ. Microbiol.* 74(8), 2360-2369. doi: AEM.02634-07 [pii]
10.1128/AEM.02634-07.

Lin, D., Rao, C.V., and Slauch, J.M. (2008). The Salmonella SPI1 type three secretion system responds to periplasmic disulfide bond status via the flagellar apparatus and the RcsCDB system. *J Bacteriol* 190(1), 87-97. doi: 10.1128/JB.01323-07.

Maurer, J.J., Brown, T.P., Steffens, W.L., and Thayer, S.G. (1998). The occurrence of ambient temperature-regulated adhesins, curli, and the temperature-sensitive hemagglutinin *tsh* among avian *Escherichia coli*. *Avian Dis.* 42(1), 106-118.

Rodriguez-Siek, K.E., Giddings, C.W., Doetkott, C., Johnson, T.J., Fakhr, M.K., and Nolan, L.K. (2005). Comparison of *Escherichia coli* Isolates Implicated in Human Urinary Tract Infection and Avian Colibacillosis. *Microbiology* 151(Pt 6), 2097-2110.

Runyen-Janecky, L.J., Reeves, S.A., Gonzales, E.G., and Payne, S.M. (2003). Contribution of the *Shigella flexneri* Sit, Iuc, and Feo iron acquisition systems to iron acquisition *in vitro* and in cultured cells. *Infect. Immun.* 71(4), 1919-1928.

Simpson, K.W., Dogan, B., Rishniw, M., Goldstein, R.E., Klaessig, S., McDonough, P.L., et al. (2006). Adherent and invasive Escherichia coli is associated with granulomatous colitis in boxer dogs. *Infect Immun* 74(8)**,** 4778-4792. doi: 10.1128/IAI.00067-06.

Supplementary Table 2: Analysis of conversion of isolates from the Clermont 2000 scheme to the Clermont 2013 scheme for human isolates - uropathogenic *E. coli* (UPEC).

		$ECOR_{2013}$								Total
		A	B1	B2	D	C	E	F	clade	NT	
$ECOR_{2000}$	A	38	2	1	0	22	0	0		0	63
	B1	0	44	0	0	0	0	0		0	44
	B2	0	0	447	4	0	1	2		2	456
	D	0	0	20	76	0	9	28		0	133
Total		38	46	468	80	22	10	30		2	696

Yellow box indicates significant difference in assignment at $P < 0.05$

Supplementary Table 3: Analysis of conversion of isolates from the Clermont 2000 scheme to the Clermont 2013 scheme for human isolates - neonatal meningitis *E. coli* (NMEC).

		$ECOR_{2013}$								Total
		A	B1	B2	D	C	E	F	clade	NT	
$ECOR_{2000}$	A	5	0	0	0	3	0	0	0	0	8
	B1	0	2	0	0	0	0	0	0	0	2
	B2	0	0	66	0	0	0	0	0	0	66
	D	0	0	0	1	0	0	7	0	0	8
Total		5	2	66	1	3	0	7	0	0	84

Yellow box indicates significant difference in assignment at $P < 0.05$

Supplementary Table 4: Analysis of conversion of isolates from the Clermont 2000 scheme to the Clermont 2013 scheme for human isolates - human vaginal *E. coli* (HVEC).

		$ECOR_{2013}$									Total
		A	B1	B2	D	C	E	F	clade	NT	
$ECOR_{2000}$	A	3	0	0	0	2	0	0	0	0	5
	B1	0	5	0	0	0	0	0	0	0	5
	B2	0	0	67	0	0	0	0	0	0	67
	D	0	0	2	5	0	0	4	0	0	11
Total		3	5	69	5	2	0	4	0	0	88

Yellow box indicates significant difference in assignment at P < 0.05

Supplementary Table 5: Analysis of conversion of isolates from the Clermont 2000 scheme to the Clermont 2013 scheme for human isolates - human fecal *E. coli* (HFEC).

		$ECOR_{2013}$									Total
		A	B1	B2	D	C	E	F	clade	NT	
$ECOR_{2000}$	A	21	0	1	2	1	0	0	1	0	26
	B1	0	32	1	0	0	0	0	0	1	34
	B2	0	1	106	1	0	0	0	0	0	108
	D	0	0	4	19	0	1	5	0	0	29
Total		21	33	112	22	1	1	5	1	1	197

Yellow box indicates significant difference in assignment at P < 0.05

Supplementary Table 6: Analysis of conversion of isolates from the Clermont 2000 scheme to the Clermont 2013 scheme for poultry isolates - avian pathogenic *E. coli* (APEC).

		ECOR2013									Total
		A	B1	B2	D	C	E	F	clade	NT	
ECOR2000	A	45	1	0	0	124	0	0	1	1	172
	B1	1	76	0	0	0	0	0	1	0	78
	B2	0	3	65	1	0	1	2	0	0	72
	D	0	4	4	22	1	14	85	0	0	130
Total		46	84	69	23	125	15	87	2	1	452

Yellow box indicates significant difference in assignment at P < 0.05

Supplementary Table 7: Analysis of conversion of isolates from the Clermont 2000 scheme to the Clermont 2013 scheme for poultry isolates - crop and gizzard *E. coli* (CGEC).

		ECOR2013									Total
		A	B1	B2	D	C	E	F	clade	NT	
ECOR2000	A	20	0	0	0	4	0	0	0	0	24
	B1	0	21	0	0	0	0	0	0	0	21
	B2	0	0	7	0	0	3	0	0	0	10
	D	0	0	0	12	0	1	12	0	0	25
Total		20	21	7	12	4	4	12	0	0	80

Yellow box indicates significant difference in assignment at P < 0.05

Supplementary Table 8: Analysis of conversion of isolates from the Clermont 2000 scheme to the Clermont 2013 scheme for poultry isolates - retail poultry *E. coli* (RPEC).

		ECOR2013									Total
		A	B1	B2	D	C	E	F	clade	NT	
ECOR2000	A	40	0	0	0	21	0	0	0	0	61
	B1	0	53	0	0	0	0	0	0	0	53
	B2	0	0	18	0	0	5	1	0	0	24
	D	0	0	1	18	0	7	36	0	0	62
Total		40	53	19	18	21	12	37	0	0	200

Yellow box indicates significant difference in assignment at $P < 0.05$

Supplementary Table 9: Analysis of conversion of isolates from the Clermont 2000 scheme to the Clermont 2013 scheme for poultry isolates - avian fecal *E. coli* (RPEC).

		ECOR2013									Total
		A	B1	B2	D	C	E	F	clade	NT	
ECOR2000	A	70	4	0	0	13	0	0	3	0	90
	B1	2	52	0	2	0	0	0	0	0	56
	B2	0	0	22	3	0	5	2	1	0	33
	D	0	1	0	9	0	4	6	0	0	20
Total		72	57	22	14	13	9	8	4	0	199

Yellow box indicates significant difference in assignment at $P < 0.05$
